# Supplementary material for: Empirical methods for controlling false positives and estimating confidence in ChIP-Seq peaks
Source: BMC Bioinformatics. 2008 Dec 5;9:523. doi: 10.1186/1471-2105-9-523 (PMC2628906; doi:10.1186/1471-2105-9-523)
Supplement: Additional File 1 — A variety of html documents from the USeq web site detailing the available applications, their best usage, output file type descriptions, command line menus, etc. [file 1471-2105-9-523-S1.zip › USeqUserGuides/JavaDoc.zip!/JavaDoc/bioroot/antibody/class-use/AntibodyBean.html]

Uses of Class bioroot.antibody.AntibodyBean


|  |  |  |
| --- | --- | --- |
| **Overview** \| **Package** \| **Class** \| **Use** \| **Tree** \| **Deprecated** \| **Index** \| **Help** | |  |
| PREV   NEXT | **FRAMES**    **NO FRAMES**     **All Classes** |  |


---


## **Uses of Class bioroot.antibody.AntibodyBean**

| Packages that use AntibodyBean | |
| --- | --- |
| **bioroot** |  |
| **bioroot.antibody** |  |

Uses of AntibodyBean in bioroot

| Methods in bioroot that return AntibodyBean | |
| --- | --- |
| `static AntibodyBean[]` | `Archive.makeAntibodyBeans(DBUtil bioRoot, UserBean userBean)`             Makes an array of all visibile AntibodyBeans |

| Methods in bioroot with parameters of type AntibodyBean | |
| --- | --- |
| `static java.util.ArrayList` | `Archive.fetchFiles(PlasmidBean[] plasmids, StrainBean[] strains, AntibodyBean[] antibodies, java.lang.String filePath, DBUtil bioRoot)`             Fetches any plasmid or strain files. |

Uses of AntibodyBean in bioroot.antibody

| Methods in bioroot.antibody that return AntibodyBean | |
| --- | --- |
| `static AntibodyBean[]` | `AntibodySpreadSheet.fetchAntibodyBeans(java.lang.String sql, DBUtil bioRoot)`             Build AntibodyBean[] from Antibody table in database. |
| `static AntibodyBean[]` | `AntibodySpreadSheet.orderAntibodies(java.lang.String featureName, UserBean userBean, AntibodyBean[] beans, DBUtil bioRoot)`             Orders antibodys, sort of nasty, can't figure another way, reflection is way too slow. |
| `static AntibodyBean[]` | `AntibodyBase.reverseAntibodyBeans(AntibodyBean[] beans)`             Reverses the order of an array of PlasmidBeans. |

| Methods in bioroot.antibody with parameters of type AntibodyBean | |
| --- | --- |
| `static void` | `NewAntibody.addAnAntibodyPrep(AntibodyBean bean, DBUtil bioRoot)`             Adds a new antibodyPrep to the AntibodyBean |
| `static int` | `AntibodySpreadSheet.addBlankLines(AntibodyBean bean, java.lang.String[] items, int k)`             Adds "" to items base on the number of preps and uses in the antibody bean. |
| `static void` | `AntibodyBase.addPlasmid(AntibodyBean bean, java.util.HashMap nameValue, DBUtil bioRoot, UserBean userBean)` |
| `static void` | `AntibodyBase.addStrain(AntibodyBean bean, java.util.HashMap nameValue, DBUtil bioRoot, UserBean userBean)` |
| `static void` | `NewAntibody.addUseDilution(AntibodyBean bean, DBUtil bioRoot, int idAntibodyPrep)`             Adds a new UseDilution to the indicated AntibodyPrep |
| `static java.lang.String` | `AntibodyModify.deleteAntibody(AntibodyBean ab, UserBean userBean, DBUtil bioRoot)`             Attempts to delete an antibody, if sucessful, returns a "" String, otherwise an error message. |
| `static int` | `AntibodyBase.deleteAntibodyPrepIndex(AntibodyBean a, DBUtil bioRoot, java.util.HashMap m)`             Scans a hashmap for an antibody prep to delete returns the index number |
| `static java.lang.String` | `AntibodyBase.deleteUseDilutionIndex(AntibodyBean a, DBUtil bioRoot, java.util.HashMap m)`             Scans a hashmap for a use dilution to delete returns the index string or null. |
| `static java.lang.String` | `AntibodySpreadSheet.fetchAntibodyTableRows(UserBean userBean, AntibodyBean[] beans, int[] startStop, DBUtil bioRoot, boolean checked, boolean allTabs)`             Returns table rows for every oligo according to a Preference, if allTabs==true, then all features are returned in a tab delimited format, otherwise its HTML. |
| `static java.lang.String` | `AntibodyBase.getAntibodyForm(AntibodyBean ab, DBUtil bioRoot, UserBean userBean)`             Returns an antibody form filled with the values in the bean. |
| `static void` | `AntibodyBase.getAntibodyPrepForms(AntibodyBean ab, DBUtil bioRoot, java.lang.StringBuffer b)` |
| `static void` | `AntibodyBase.getAntibodyPrepReports(AntibodyBean ab, DBUtil bioRoot, java.lang.StringBuffer b, boolean commonForm)` |
| `static java.lang.String` | `AntibodyBase.getAntibodyReport(AntibodyBean ab, DBUtil bioRoot, UserBean user, boolean commonForm)`             Returns an antibody report filled with the values in the bean. |
| `static void` | `NewAntibody.loadAntibodyBean(java.util.HashMap nameValue, DBUtil bioRoot, AntibodyBean ab, javax.servlet.http.HttpServletRequest request)` |
| `static void` | `NewAntibody.loadAntibodyPrepBeans(java.util.HashMap nameValue, DBUtil bioRoot, AntibodyBean ab, javax.servlet.http.HttpServletRequest request)` |
| `static int` | `AntibodyBase.newUseDilutionIndex(AntibodyBean a, DBUtil bioRoot, java.util.HashMap m)`             Scans a hashmap for a newUseDilution selection, returns the index number |
| `static AntibodyBean[]` | `AntibodySpreadSheet.orderAntibodies(java.lang.String featureName, UserBean userBean, AntibodyBean[] beans, DBUtil bioRoot)`             Orders antibodys, sort of nasty, can't figure another way, reflection is way too slow. |
| `static void` | `AntibodyBase.removePlasmid(AntibodyBean bean, java.util.HashMap nameValue, DBUtil bioRoot)` |
| `static void` | `AntibodyBase.removeStrain(AntibodyBean bean, java.util.HashMap nameValue, DBUtil bioRoot)` |
| `static AntibodyBean[]` | `AntibodyBase.reverseAntibodyBeans(AntibodyBean[] beans)`             Reverses the order of an array of PlasmidBeans. |
| `static boolean` | `AntibodyModify.subtractAndSetAntibodyBeans(AntibodyBean[] beans, javax.servlet.http.HttpSession session)`             Subtracts a bean from the AntibodyBean[] and resets it in antibodyBeansToEdit |
| `static java.lang.String` | `AntibodyModify.updateAntibody(AntibodyBean bean, UserBean userBean, DBUtil bioRoot)`             Attempts to update a AntibodyBean in the database, only checks that the name is unique if it has changed. |
| `static java.lang.String` | `AntibodyModify.updateAntibodyPreps(AntibodyBean bean, DBUtil bioRoot)`             Attempts to update the AntibodyPreps and UseDilutions in the database. |

---


|  |  |  |
| --- | --- | --- |
| **Overview** \| **Package** \| **Class** \| **Use** \| **Tree** \| **Deprecated** \| **Index** \| **Help** | |  |
| PREV   NEXT | **FRAMES**    **NO FRAMES**     **All Classes** |  |


---
